# An assessment of gender inequitable norms and gender-based violence in South Sudan: a community-based participatory research approach

**DOI:** 10.1186/1752-1505-7-4

**Published:** 2013-03-06

**Authors:** Jennifer Scott, Sarah Averbach, Anna Merport Modest, Michele R Hacker, Sarah Cornish, Danielle Spencer, Maureen Murphy, Parveen Parmar

**Affiliations:** 1 Harvard Humanitarian Initiative, Cambridge, Massachusetts (MA), USA; 2 Department of Medicine, Division of Women’s Health, Brigham and Women’s Hospital, Boston, MA, USA; 3 Department of Obstetrics and Gynecology, Beth Israel Deaconess Medical Center, Boston, MA, USA; 4 Department of Emergency Medicine, Division of International Health and Humanitarian Programs, Brigham and Women’s Hospital, Boston, MA, USA; 5 Harvard School of Public Health, Department of Epidemiology, Boston, MA, USA; 6 American Refugee Committee, Juba, South Sudan

**Keywords:** South Sudan, Conflict, Gender norms, Gender equality, Gender-based violence, Gender Equitable Men scale

## Abstract

**Background:**

Following decades of conflict, South Sudan gained independence from Sudan in 2011. Prolonged conflict, which included gender-based violence (GBV), exacerbated gender disparities. This study aimed to assess attitudes towards gender inequitable norms related to GBV and to estimate the frequency of GBV in sampled communities of South Sudan.

**Methods:**

Applying a community-based participatory research approach, 680 adult male and female household respondents were interviewed in seven sites within South Sudan in 2009–2011. Sites were selected based on program catchment area for a non-governmental organization and respondents were selected by quota sampling. The verbally-administered survey assessed attitudes using the Gender Equitable Men scale. Results were stratified by gender, age, and education.

**Results:**

Of 680 respondents, 352 were female, 326 were male, and 2 did not provide gender data. Among respondents, 82% of females and 81% of males agreed that ‘a woman should tolerate violence in order to keep her family together’. The majority, 68% of females and 63% of males, also agreed that ‘there are times when a woman deserves to be beaten’. Women (47%) were more likely than men (37%) to agree that ‘it is okay for a man to hit his wife if she won’t have sex with him’ (p=0.005). Agreement with gender inequitable norms decreased with education. Across sites, 69% of respondents knew at least one woman who was beaten by her husband in the past month and 42% of respondents knew at least one man who forced his wife or partner to have sex.

**Conclusion:**

The study reveals an acceptance of violence against women among sampled communities in South Sudan. Both women and men agreed with gender inequitable norms, further supporting that GBV programming should address the attitudes of both women and men. The results support promotion of education as a strategy for addressing gender inequality and GBV. The findings reveal a high frequency of GBV across all assessment sites; however, population-based studies are needed to determine the prevalence of GBV in South Sudan. South Sudan, the world’s newest nation, has the unique opportunity to implement policies that promote gender equality and the protection of women.

## Background

Since Sudan gained independence from British rule in 1956, Sudanese communities have experienced ongoing civil war, tribal conflict, and displacement. South Sudan has been home to some of the most extreme violence and insecurity within what was once a united Sudan. The 2005 Comprehensive Peace Agreement (CPA) granted the South a six-year period of autonomy to be followed by a referendum. The January 2011 referendum favored secession of South Sudan and resulted in the independence of South Sudan from Sudan on 9 July 2011 [1].

During periods of protracted conflict, as in South Sudan, gender-based violence (GBV) is one of the most pervasive forms of violence [2,3]. The United Nations defines GBV as “any act that results in, or is likely to result in physical, sexual, or psychological harm or suffering to women, including threats of such acts, coercion or arbitrary deprivation of liberty, whether occurring in public or in private life” and can include intimate partner violence (IPV), sexual violence, and rape among other forms of GBV [4,5]. In South Sudan, prolonged conflict has exacerbated and created new security risks for women including disruption of community and family structures, presence of arms, weakened legal and security institutions, and heightened tensions related to displacement [6,7].

The extent of GBV occurring in South Sudanese communities, both during the war and following the 2005 CPA, has not been systematically evaluated. In regard to conflict-related GBV, a 2004 survey of South Sudanese refugees living in Uganda revealed that 33% had witnessed the rape of a woman, 10% had been raped, 9% had traded sex for food or security, and 8% reported sexual slavery or forced prostitution [8]. A 2011 report from South Sudan reported that 25% of all women surveyed were involved in war-related activities, including traveling with arms and food and providing sexual services [9].

In regard to GBV occurring within households and communities, numerous incident and anecdotal reports indicate GBV to be a widespread problem in South Sudan. In a 2009 study, 41% of respondents reported they had experienced GBV in the past year and 29% reported knowing someone who had experienced GBV in the past year. The most commonly reported forms of GBV included physical violence (47%), psychological violence (44%), economic violence (30%), and sexual violence (13%) [10]. Evidence collected from interviews in a 2011 report revealed that 59% of surveyed women reported GBV in the home and 19% reported GBV in the community [9]. Reports from human rights groups indicate that there is evidence of extensive domestic violence, sexual harassment, and sexual assault in South Sudan [11].

While studies and reports indicate GBV to be a widespread problem in South Sudan, there are limited data about how gender dynamics and gender inequities contribute to GBV in South Sudanese communities. GBV is rooted in gender inequality and GBV prevention and response interventions are encouraged to address underlying gender dynamics [12]. During humanitarian crises, GBV increases due to an acceptance of violence against women in communities, breakdown in law and order, the use of GBV as a weapon of war, and polarization of gender roles [13]. Gender inequality data specific to South Sudan are limited given its recent independence, but development indices for Sudan, including the Social Institutions and Gender Index, the Gender Inequality Index, and the Gender Parity Index indicate low equality between women and men in terms of reproductive health, empowerment, education, and employment [14,15]. The ratified 2011 Transitional Constitution of the Republic of South Sudan includes specific gender priorities [16], and the country is working towards achieving Millennium Development Goal 3 by 2015: to promote gender equality and empower women [17].

The primary objectives of this study were to provide data on gender inequitable norms related to GBV and to understand the effects of gender, age, and education on these attitudes and beliefs. A secondary objective was to estimate the frequency of GBV in the communities identified to be priority areas for GBV programming in South Sudan. The American Refugee Committee (ARC), a non-governmental organization, conducts GBV prevention and response programming in five states and seven counties in South Sudan. The assessments, performed as part of ARC programming in 2009–2011, aimed to provide baseline GBV data in terms of the 2011 independence and to inform future gender and protection policies for South Sudan.

## Methods

Applying a community-based participatory research approach, assessments were conducted as part of ARC programming in seven counties of South Sudan during 2009–2011, prior to the country’s independence. (Table 1) The violence assessment was part of a larger assessment as described below.

**Table 1:** Assessment sites in South Sudan

**Site of Study**	**Aweil**	**Kwajok**	**Lainya**	**Malakal**	**Morobo**	**Ronyi**	**Wau**
**County**	Aweil Centre	Gogrial West	Lainya	Malakal	Morobo	Yei	Wau
**State**	Northern Bahr El Ghazal	Warrap	Central Equatoria	Upper Nile	Central Equatoria	Central Equatoria	Western Bahr El Ghazal
**Population**	41,827	243,921	89,315	126,483	103,603	201,443	151,320
**Population (Males / Females)**	22199 (53%) 19628 (47%)	116994 (48%) 126927 (52%)	47424 (53%) 41891 (47%)	66707 (53%) 59776 (47%)	52976 (51%) 50627 (49%)	105165 (52%) 96278 (48%)	80781 (53%) 70539 (47%)
**Year of baseline evaluation**	2011	2011	2009	2011	2009	2009	2011
**Sample size of baseline evaluation**	100	94	100	100	100	100	102

Table compiled by authors. Population data obtained from Statistical Yearbook for South Sudan 2010 [18]. All assessments were conducted prior to South Sudan’s independence on 9 July 2011.

### ARC assessment in South Sudan

#### Sampling

The assessment sites were selected based on the projected catchment area of ARC programs. The geographic area targeted for ARC programming was mapped and divided into zones of approximately equal population sizes. If a formal map of the community was not available, the community steering committee and/or community interviewers were asked to plot a map of the town. Population data were obtained from the 2008 census to estimate population and demographic distribution for each site [18]. The community steering committee and/or community interviewers were asked to provide input regarding population and demographic distribution.

In each assessment site, pairs of interviewers were assigned a zone. A random start point was assigned for the zone and interviewers traversed the zone, selecting households at a pre-determined random interval (i.e., every 5^th^ household). For the purposes of these assessments, a household was defined as a group of people who usually live and eat together. If a household member was not available on the first attempt, the interviewers were instructed to proceed to the next household, according to the pre-determined interval. Interviewers were not asked to return to a household if a respondent was unavailable on the first attempt.

A sample size of 100 men and women was pre-determined for each assessment site. Quota sampling was applied and on each survey day the estimated age and gender distribution of the site was used to determine the number of male and female respondents needed in each of the following two age strata: 18–35 years and >35 years.

If the intended respondent (i.e. older man) was unavailable at the first household, the interviewers were asked to proceed to the next household, according to the pre-determined interval, until they found a respondent who met the designated criteria. Only one individual was interviewed per household. Upon selection of the respondent at the household, the survey was introduced and verbal informed consent was obtained. The respondents were informed that they would not receive compensation for their responses and that their participation was voluntary. The assessment was intended to survey adults 18 years of age or older; however, in several sites, the community steering committee wanted to understand the experiences of those under age 18. For respondents aged 12–18 years, verbal consent was obtained from an adult household member and verbal assent was obtained from the minor.

Records of refusals or lack of availability were not kept in all sites. For the 2011 assessment in Wau, there was a response rate of 96% (102/106). Team leaders for the other sites reported a similar response rate; however, exact response rates are not available for those sites.

#### Instrument

The survey instrument consisted of 52 questions, including 7 questions on demographics, 28 questions on attitudes and beliefs regarding gender inequitable norms, 6 questions on information sharing, 10 questions on frequency of GBV and traditional practices in the community, and 1 question about the empowerment of women. At each assessment site, the community steering committee had the opportunity to determine up to 6 additional questions; thus, the surveys ranged in length from 52–58 questions. Of the 28 questions assessing attitudes and beliefs regarding gender inequitable norms, 17 were adapted from the Gender Equitable Men (GEM) scale inequitable subscale (explained below) and the remaining questions on gender inequitable norms were determined at the outset of the study in collaboration with a community steering committee. The survey instrument was written in English and translated into Arabic. It was also back translated for accuracy. The interviewers verbally administered the survey to the respondents in Arabic in the majority of sites or in the regional language when necessary. The survey questions, including the GEM scale questions, were divided into to the following domains: violence, sexual relationships, reproductive health, traditional practices, and gender roles and dynamics. A copy of the survey instrument in English and in Arabic is available on request from the authors.

#### Gender Equitable Men scale

The GEM scale [19,20] is a 24-item questionnaire developed to measure attitudes towards gender equitable norms. It consists of a list of statements about women’s and men’s roles related to domestic and sexual life and intimate relationships, including 17 statements in an inequitable subscale and 7 in an equitable subscale. Respondents are asked to indicate whether they ‘agree’, ‘partially agree’, or ‘disagree’ with the statements using a three-point scale. Only the questions assessing violence against women are presented in this paper.

A gender equitable man is defined as a man who is respectful to women, who believes that men and women should have equal rights, and who shares responsibility in the household. A gender equitable man is opposed to violence against women [19]. The GEM scale was developed based on qualitative research on gender norms with young men in low-income settings in Brazil but has been validated in India and Kenya and has been used in Ethiopia, Uganda, and Nicaragua [21]. Studies have demonstrated that agreement with inequitable statements correlates with physical and sexual violence in surveyed communities [22]. The GEM scale has not been validated specifically for use in conflict and post-conflict settings. The GEM scale is not designed to assess a gender equitable woman.

#### Interviewers

Trained male and female South Sudanese interviewers verbally administered the survey. For each assessment site, the interviewers were selected from that region and were fluent in the local dialect and customs. In most cases, interviewers worked for ARC or a local organization and had prior interviewing experience. Each site conducted a two-day training focused on the survey instrument, sampling, and community mapping. The training was conducted in Arabic, and, where applicable, also in English. In each site, a South Sudanese ARC staff member and an international ARC GBV Program Coordinator conducted the training.

#### Ethics

ARC has permission from the government of the Republic of South Sudan to conduct its programming and assessments in South Sudan. Within each designated sampling zone, permission was obtained from the local village chief or elder to conduct the survey in that zone. Ethics approval was granted from Harvard School of Public Health. ARC senior management also conducted a review of the proposed study. ARC coordinated basic care, GBV support services, and referral if requested by a respondent or if determined necessary by the interviewer.

Verbal informed consent was obtained from each survey respondent. Ethical review limited the final data analysis to those aged 18 and older. The survey was administered within the household in as private a setting as possible. No names or identifying personal information were collected in the course of the study. Data were kept anonymous and were stored securely with ARC staff at all times. The protection of respondents and interviewers and confidentiality were priorities. Up-to-date security information was obtained daily and transmitted to the research team.

### Violence assessment

The data regarding violence are presented in this paper. Responses of women and men were compared as well as the responses across age strata (≤35 years and >35 years) and education strata (no education and any education). For purposes of analysis and comparison across groups, the responses ‘agree’ and ‘partially agree’ were combined together into one variable ‘agree’. Only the responses of those ages 18 and older were included in the analysis.

### Statistical analysis

Statistical analysis was performed using SAS® 9.3 statistical software (SAS institute Inc., Cary, NC). Data are reported as mean and standard deviation, median and interquartile range (IQR) or proportion depending on data type and distribution. Parametric and non-parametric tests were used as appropriate. The chi-squared test was applied for categorical data. All tests were two-sided and p-values <0.05 were considered statistically significant. We used log binomial regressions to control for any potential confounders. Variables were considered confounders if they changed the risk ratio by more than 10%.

## Results

A total of 697 respondents were interviewed; however, after excluding respondents less than 18 years of age, 680 interviews were analyzed.

### Demographics

Of the 680 respondents, 352 (52%) were women and 326 (48%) were men, and 2 individuals did not have gender data recorded. The median age of all respondents was 34.0 years (25.0-45.0). The majority (70%) of respondents were married. Respondents self-reported a median of 5.0 years (0.0-9.0) of formal education, with the majority (56%) reporting a low literacy level. One-third of the surveyed population reported being able to ‘read easily.’ However, men had more years of education than women (p<0.0001) and a higher reading level (p<0.0001). The majority (62%) of respondents reported to be of Christian faith and represented the major ethnic groups of Dinka, Kakwa, and Shilluk, among others. The self-reported ethnicity varied by region surveyed. Of note, for two sites (Lainya 2009 and Ronyi 2009) data on religion and ethnicity were collected and entered; however, the original coding key for these two variables was not available, and thus is reported as ‘missing’ in the final analysis. Demographic characteristics are reported in Table 2.

**Table 2:** Baseline respondent characteristics

**Characteristic**	**All respondents n=680**	**Male† n=326**	**Female† n=352**	P*
**Age (years)—median (IQR)**	34.0 (25.0-45.0)	35.0 (25.0-45.0)	33.0 (25.0-44.5)	0.21
**Marital Status—n (%)**				<0.0001
**Single**	120 (17.7)	84 (25.8)	36 (10.2)	
**Married**	476 (70.0)	210 (64.4)	264 (75.0)	
**Widowed or divorced**	39 (5.7)	7 (2.2)	32 (9.1)	
**Unknown**	45 (6.6)	25 (7.7)	20 (5.7)	
Religion**—n (%)				0.86
**Christian**	419 (61.6)	200 (61.4)	217 (61.6)	
**Muslim**	40 (5.9)	20 (6.1)	20 (5.7)	
**Other**	8 (1.2)	5 (1.5)	3 (0.9)	
**Unknown**	213 (31.3)	101 (31.0)	112 (31.8)	
Ethnicity**—n (%)				0.43
**Dinka**	209 (30.7)	100 (30.7)	107 (30.4)	
**Kakwa**	77 (11.3)	39 (12.0)	38 (10.8)	
**Shilluk**	48 (7.1)	17 (5.2)	31 (8.8)	
**Other**	141 (20.7)	72 (22.1)	69 (19.6)	
**Missing**	205 (30.2)	98 (30.1)	107 (30.4)	
**Literacy—n (%)**				<0.0001
**Reads easily**	228 (33.5)	141 (43.2)	87 (24.7)	
**Reads with difficulty**	161 (23.7)	75 (23.0)	86 (24.3)	
**Not at all**	222 (32.6)	80 (24.5)	140 (39.8)	
**Unknown**	69 (10.1)	30 (9.2)	39 (11.1)	
**Education (years)—median (IQR)**	5.0 (0.0-9.0)	7.0 (2.0-10.0)	4.0 (0.0-8.0)	<0.0001

IQR=Interquartile range

†2 participants were missing gender data;

*Comparison is between males and females;

**Data missing for Lainya 2009, Ronyi 2009.

### Attitudes and beliefs: violence domain

Among respondents, 82% of women and 81% of men agreed with the statement ‘a woman should tolerate violence in order to keep her family together’. (Table 3) The majority of respondents, 68% of women and 63% of men, also agreed that ‘there are times when a woman deserves to be beaten’. Similar levels of agreement were seen in both age groups. When the responses to the violence domain questions were compared according to education, respondents who reported no education (73.6%) were more likely than those who reported any education (62.2%) to agree that ‘there are times when a woman deserves to be beaten’ (p=0.01) (Table 4).

**Table 3:** Violence domain stratified by gender and age

**Survey question**	**Response**	**Male (n=326)**	**Female (n=352)**	P*		**Age ≤35 yrs (n=367)**	**Age >35 yrs (n=313)**	P*
***A woman should tolerate violence in order to keep her family together***	**Agree**	263 (80.7)	289 (82.1)	0.53	**Agree**	304 (82.8)	250 (79.9)	0.42
	**Disagree**	61 (18.7)	59 (16.8)		**Disagree**	61 (16.2)	59 (18.9)	
	**Missing**	2 (0.6)	4 (1.1)		**Missing**	2 (0.5)	4 (1.3)	
***There are times when a woman deserves to be beaten***	**Agree**	205 (62.9)	240 (68.2)	0.13	**Agree**	250 (68.1)	197 (62.9)	0.21
	**Disagree**	120 (36.8)	110 (31.3)		**Disagree**	117 (31.9)	113 (36.1)	
	**Missing**	1 (0.3)	2 (0.6)		**Missing**	0 (0.0)	3 (1.0)	
***It is okay for a man to hit his wife if she won’t have sex with him***	**Agree**	121 (37.1)	167 (47.4)	0.005	**Agree**	151 (41.1)	138 (44.1)	0.39
	**Disagree**	202 (62.0)	180 (51.1)		**Disagree**	213 (58.4)	170 (54.3)	
	**Missing**	3 (0.9)	5 (1.4)		**Missing**	3 (0.8)	5 (1.6)	
***If someone insults a man, he should defend his reputation, with force if he has to***	**Agree**	166 (50.9)	209 (59.4)	0.01	**Agree**	198 (54.0)	179 (57.2)	0.26
	**Disagree**	158 (48.5)	134 (38.1)		**Disagree**	166 (45.2)	126 (40.3)	
	**Missing**	2 (0.6)	9 (2.6)		**Missing**	3 (0.8)	8 (2.6)	
***If a woman is beaten by her husband, she should not tell anyone***	**Agree**	181 (55.5)	196 (55.7)	0.94	**Agree**	190 (51.8)	189 (60.4)	0.03
	**Disagree**	143 (43.9)	153 (43.5)		**Disagree**	174 (47.4)	122 (39.0)	
	**Missing**	2 (0.6)	3 (0.9)		**Missing**	3 (0.8)	2 (0.6)	
***If a girl or woman is raped, it is better for her to keep it to herself***	**Agree**	106 (32.5)	110 (31.2)	0.72	**Agree**	126 (34.3)	91 (29.1)	0.18
	**Disagree**	216 (66.3)	238 (67.6)		**Disagree**	239 (65.1)	216 (69.0)	
	**Missing**	4 (1.2)	4 (1.1)		**Missing**	2 (0.5)	6 (1.9)	

*Missing data category not included in comparisons

**Table 4:** Violence domain stratified by education level

**Survey question**	**Response**	**No education (n=159)**	**Any education (n=444)**	P*
***A woman should tolerate violence in order to keep her family together***	**Agree**	140 (88.1)	357 (80.4)	0.052
	**Disagree**	19 (12.0)	82 (18.5)	
	**Missing**	0 (0.0)	5 (1.1)	
***There are times when a woman deserves to be beaten***	**Agree**	117 (73.6)	276 (62.2)	0.01
	**Disagree**	42 (26.4)	167 (37.6)	
	**Missing**	0 (0.0)	1 (0.2)	
***It is okay for a man to hit his wife if she won’t have sex with him***	**Agree**	95 (59.8)	168 (37.8)	<0.0001
	**Disagree**	64 (40.3)	271 (61.0)	
	**Missing**	0 (0.0)	5 (1.1)	
***If someone insults a man, he should defend his reputation, with force if he has to***	**Agree**	96 (60.4)	232 (52.3)	0.07
	**Disagree**	61 (38.4)	207 (46.6)	
	**Missing**	2 (1.3)	5 (1.1)	
***If a girl or woman is raped, it is better for her to keep it to herself***	**Agree**	58 (36.5)	141 (31.8)	0.26
	**Disagree**	99 (62.3)	300 (67.6)	
	**Missing**	2 (1.3)	3 (0.7)	
***If a woman is beaten by her husband, she should not tell anyone***	**Agree**	95 (59.8)	227 (51.1)	0.051
	**Disagree**	62 (39.0)	214 (48.2)	
	**Missing**	2 (1.3)	3 (0.7)	

*Missing data category not included in comparisons.

While fewer respondents agreed that ‘it is okay for a man to hit his wife if she won’t have sex with him’, women (47.4%) were more likely than men (37.1%) to agree with this statement (p=0.005). Furthermore, those respondents who reported no education (59.8%) were more likely than those who reported any education (37.8%) to agree with this statement (p=<0.0001). Women (59.4%) were more likely than men (50.9%) to agree ‘if someone insults a man, he should defend his reputation with force if he has to’ (p=0.01).

In response to the statement, ‘if a woman is beaten by her husband, she should not tell anyone’, the majority of women (56%) and men (56%) agreed. Those older than 35 years of age were more likely than younger respondents to agree that a woman should not disclose this type of violence (p=0.03). More respondents who reported no education (59.8%) agreed that ‘if a woman is beaten by her husband, she should not tell anyone’ than those who reported any education (51.1%); however, this result was of borderline statistical significance (p=0.051). In contrast, fewer respondents agreed that ‘if a woman is raped, it is better for her to keep it to herself’ and there were no differences by gender, age, or years of education.

We conducted multivariate analyses to assess whether one gender was more likely to agree with selected statements in the violence domain controlling for age and education, but we did not find a difference.

### Frequency of GBV in sampled communities

Table 5 presents reported estimates of GBV in each sampled community and regional trends in the frequency of GBV. When the data are aggregated across sites, 69% of respondents knew at least one woman who was beaten by her husband in the past month. When asked how many men forced their wives or partners to have sex when they didn’t want to, 42% of respondents knew of at least one occurrence in the past month. When asked how many men got drunk and hurt their wives or partners, 80% of respondents knew of at least one occurrence in the past month.

**Table 5:** Frequency of reported gender-based violence by site in South Sudan

	**All sites n=680**	**Aweil n=100**	**Kwajok n=90**	**Lainya n=98**	**Malakal n=100**	**Morobo n=93**	**Ronyi n=98**	**Wau n=101**
***In the past month…of your friendsand family, how many women were beaten by their husbands*—n (%)**								
**0**	140 (20.6)	19 (19.0)	12 (13.3)	10 (10.2)	35 (35.0)	28 (30.1)	19 (19.4)	17 (16.8)
**1-9**	300 (44.1)	56 (56.0)	59 (65.6)	22 (22.4)	31 (31.0)	43 (46.2)	59 (60.2)	30 (29.7)
**10 or more**	167 (24.6)	25 (25.0)	16 (17.8)	35 (35.7)	31 (31.0)	18 (19.4)	19 (19.4)	23 (22.8)
**Unknown**	73 (10.7)	0 (0.0)	3 (3.3)	31 (31.6)	3 (3.0)	4 (4.3)	1 (1.1)	31 (30.7)
***In the past month…of your friends and family, how many men forced their wives/partners to have sex when the woman didn’t want to?—*n (%)**								
**0**	312 (45.9)	38 (38.0)	49 (54.4)	38 (38.8)	54 (54.0)	55 (59.1)	53 (54.1)	25 (24.8)
**1-9**	185 (27.2)	35 (35.0)	31 (34.4)	23 (23.5)	31 (31.0)	17 (18.3)	23 (23.5)	25 (24.8)
**10 or more**	103 (15.2)	26 (26.0)	7 (7.8)	34 (34.7)	12 (12.0)	9 (9.7)	3 (3.1)	12 (11.9)
**Unknown**	80 (11.8)	1 (1.0)	3 (3.3)	3 (3.1)	3 (3.0)	12 (12.9)	19 (19.4)	39 (38.6)
***In the past month…how many men do you know who got drunk and then hurt their wives or partners?—*n (%)**								
**0**	101 (14.9)	19 (19.0)	5 (5.6)	10 (10.2)	15 (15.0)	19 (20.4)	23 (23.5)	10 (9.9)
**1-9**	226 (33.3)	30 (30.0)	29 (32.2)	38 (38.8)	31 (31.0)	34 (36.6)	45 (45.9)	19 (18.8)
**10 or more**	317 (46.6)	51 (51.0)	53 (58.9)	48 (49.0)	51 (51.0)	36 (38.7)	23 (23.5)	55 (54.5)
**Unknown**	36 (5.3)	0 (0.0)	3 (3.3)	2 (2.0)	3 (3.0)	4 (4.3)	7 (7.1)	16 (15.8)

## Discussion

Our findings reveal an acceptance and high frequency of violence against women in the sampled communities of South Sudan.

### Gender-based violence in South Sudan

There is an overwhelming acceptance of violence against women, by both women and men, in the surveyed communities of South Sudan. The majority of respondents, regardless of gender, age, or years of education, agree that there are times when a woman deserves to be beaten and that a woman should tolerate violence in order to keep her family together. A longitudinal study of GBV customary court cases in South Sudan cited many court proceedings in which wife beating was considered acceptable if there was a reason noted for the wife beating. The community and the customary courts also prioritize preservation of the marriage and the family unit, often to the detriment of women, and thus women are encouraged to tolerate violence within the family [23].

Women and men responded similarly to inequitable statements regarding violence against women. While the GEM scale assesses attitudes and beliefs towards violence, it does not allow for conclusions to be drawn about why such acceptance of violence exists in the sampled communities nor does it explain why women support inequitable norms against women. A 2003–2007 meta-analysis of IPV using Demographic and Health Survey data in 17 Sub-Saharan African countries found that women were more likely to justify wife beating than men and that sex disparities in attitudes towards IPV increased with the practice of polygamy [24]. While our study does not assess polygamy, the 2006 Sudan Household Health Survey (SHHS) found that 27.5% of women in South Sudan were in polygamous unions [14]. The meta-analysis also found that the magnitude of sex disparities in IPV attitudes decreased with increasing adult male and female literacy rates, gender development index, gross domestic product, and human development index [24]. Among the respondents in our study, men had significantly more years of education than women, and when we compared responses across education strata, those respondents who reported no education were more likely to agree with inequitable norms regarding violence. Ethnic and cultural traditions may also reinforce this acceptance of violence against women. However, due to the numerous ethnic groups represented in our sample, it is not possible to determine the relationship between ethnicity and agreement with inequitable norms. In a 2008 report, it was noted that despite individual differences in tribal customs and traditions among ethnic groups of South Sudan, violence against women was tolerated and accepted among the major ethnic groups [23].

 The assessments provide data about estimated frequency of violence within intimate partnerships. When data are aggregated across sites, the majority of respondents knew at least one woman who was beaten by her husband (69%) and at least one man who got drunk and hurt his wife (80%). Given that these data are only estimates based on social or community networks they cannot be directly compared to estimates of IPV in other studies. Estimates of IPV in South Sudan vary between 10-59% [8-10,25]. Our study did not ask an individual to recount his or her personal experience of violence, but rather asked the respondents to provide an estimate of community frequency. There is a need for population-based data to more completely understand the prevalence of GBV in South Sudan.

Violence within intimate partnerships can include physical violence and forced sexual intercourse. Data from the 2011 UNHCR *et al.* report revealed that 7% of female respondents in South Sudan admitted to being physically forced to have sexual intercourse with their husbands [9]. However, the report mentions that the quantitative result was lower than what was reported in qualitative interviews. A 2009 mixed-methods study in South Sudan reported that sexual violence was perpetrated by husbands in 17% of cases or a distant relative in 15% of cases [10]. In our study, when data were aggregated across sites, 42% of respondents knew of at least one man who forced his wife to have sex. Furthermore, 51% of women and 45% of men agreed that a married woman should have sex with her husband even if she does not want to. Again, the estimates in our study were based on social or community networks and cannot be directly compared to other studies. Estimates about forced intercourse, or rape, within intimate partnerships may vary due to reporting bias or a lack of recognition in the community that rape can occur within an intimate partnership or marriage. The customary laws regarding rape in South Sudan often reflect the culture and do not include rape in the context of marriage. In order to address GBV in all of its forms, the definition of GBV should include violence within intimate partnerships, including forced intercourse, and the laws must appropriately recognize all forms of GBV.

### Opening the dialogue about gender-based violence in communities

Related to an overall tolerance of violence is a general lack of disclosure or discussion about violence among the respondents. The majority of respondents, including women and men of all ages, agreed that if a woman is beaten by her husband, she should not tell anyone. This silence around GBV was also reported in a 2009 study in South Sudan in which half of the surveyed sexual violence survivors did not disclose the incident [10]. What is difficult to discern from our data is why women and men agree that violence should not be disclosed. It is possible that violence is not disclosed due to guilt, shame, and stigma that have been noted in other GBV studies in Africa [3]. It is also possible that acceptance of violence between men and women is considered an acceptable practice within the household.

 There appears to be a spectrum of violence within sampled communities that is tolerated and discussed. While respondents agreed that violence between a man and woman in a marriage should not be disclosed, when questioned about rape in general, the majority of respondents (68% of women and 66% of men) supported disclosure. The use of the term ‘rape’ in this survey question was likely interpreted by the respondents as forced sexual acts occurring outside of an intimate partnership. This finding about disclosure suggests that there is a threshold of violence at which point it is recognized by the community that survivors should seek further assistance. It could speak to the success of prior and ongoing GBV sensitization campaigns about rape. It suggests that in certain circumstances people are willing to speak about rape, and it is important for GBV response programs to determine which acts of GBV are being reported in a community. Furthermore, it is important to know to whom and where these acts of violence are reported and to engage the appropriate actors to ensure that impunity does not prevail. Finally, the difference in attitudes towards disclosure of violence may reflect the cultural and legal context of South Sudan in that forced intercourse within a marriage is not considered as rape. Future GBV programming, policies, and laws, should emphasize that GBV includes rape within an intimate partnership and marriage and ensure that appropriate legal, medical, and psychosocial resources are in place for GBV survivors.

### Implications for GBV programming and research in South Sudan

These data provide estimates of GBV in sampled communities that can inform GBV programming and research in South Sudan. Understanding and addressing this tolerance of violence, with specific attention to the attitudes of women and the younger generation, will be central to effective GBV prevention and response programming. Given the acceptance of violence among the younger respondents in many of the questions, awareness campaigns aimed at the younger generation, including educational campaigns within schools, may promote a change in attitudes and beliefs. The results also indicate that education decreases acceptance of inequitable norms; thus, supporting promotion of education as a strategy for addressing gender inequality and GBV. Furthermore, GBV programs often focus on changing the attitudes of men; however, our data suggest that it is just as important, if not more important, to also address women’s views about violence and tolerance of violence. Future research could investigate how both women and men reinforce inequitable norms and practices within families and communities. Finally, further research on GBV is needed, especially population-based assessments, to understand the prevalence of GBV in South Sudanese communities.

### GBV in a post-conflict state

This study assesses attitudes towards gender inequitable norms regarding GBV and estimates the frequency of GBV in sampled South Sudanese communities in 2009–2011; however, it does not aim to assess GBV occurring during the war or as a result of the protracted conflict. There are multiple proposed contributors to the acceptance and occurrence of violence within a community. As in other post-conflict states, there is the possibility that the violence against women associated with the conflict has now been integrated into the community [26]. It is also possible that the protracted conflict has eroded family and community structures that allow violence to escalate [23]. However, experts agree that there is a continuum of violence and that violence against women in wartime is a reflection of violence against women in peacetime [27]. It is likely that gender inequality is at the root of violence within relationships, households, and communities in South Sudan. As the world’s newest nation, South Sudan has the unique opportunity to implement policies that promote gender equality and to address GBV in its communities.

#### Limitations

The findings of this study reflect the experience of selected women and men within the surveyed communities of South Sudan, but the methodology does not allow for the findings to be generalizable to the entire population of South Sudan, nor does it allow for conclusions to be drawn about prevalence. The assessments applied purposive sampling stratified by gender and age, which may only approximate a representative sample. The assessments were done as part of ARC’s programming and while every attempt was made to have consistent methodology applied at each site, variations by site, as described above, occurred in the course of doing fieldwork. Records were not kept for each site in terms of number of households accessed or response rates during the course of sampling, although the interviewers reported that there were few skipped households and few who declined to be interviewed. It is possible that the attitudes of those who were at home during the time of the study are different than the attitudes of those respondents who were unavailable or who declined to participate.

 The survey instrument included questions about sensitive community issues, and it is possible that responses could be exaggerated and underreported depending on whether the respondent perceived some gain or disadvantage to responding in a certain manner. It is possible that the gender of the interviewer influenced responses about gender-related questions. The original intention of the questions may be affected by written translation into Arabic and verbal administration in a local dialect. The questions that asked respondents to estimate the frequency of GBV events are limited in that they only reflect social or community networks of unknown size, could represent the same person, and cannot be compared to data from other studies. While the use of the GEM scale has been validated and used in similar countries, South Sudan’s history and ethnic diversity are unique. The questions are purposely designed to include strong statements of beliefs, and these questions may not be culturally appropriate and may not reflect a person’s true belief given that they are asked to agree, partially agree, or disagree with these constructed statements. The questions also do not allow for further analysis of why men and women hold certain views. Finally, the questions are written to assess a ‘gender-equitable man’, but they are not designed to assess a ‘gender-equitable woman.’ The questions do not equitably assess the experience of violence against both men and women and do not acknowledge that both men and women can be perpetrators of violence.

 In terms of sampling, the same methodology was applied at each site; however, each team was unique and could not be standardized across sites. It is possible that the varied levels of training and experience of the interviewers could affect the outcome of the study. Furthermore, within each team, there was an individual responsible for data entry. The databases were not consistently coded across sites; thus, some variables, such as religion and ethnicity, for two sites could not be included in the analysis. Aside from these two variables, the remaining data was coded consistently across sites in the final database.

 While the dynamic security situation is a daily part of programming in a post-conflict state, the effect of the political climate on the responses should be considered. For example, some respondents during the course of the study in Wau questioned whether the trained interviewers were actually government officials from northern Sudan conducting questionnaires under the auspices of a non-governmental organization. Given the renewed conflict in some of the border areas of South Sudan and the recent independence, it is possible that heightened caution within communities affected responses among those surveyed.

## Conclusion

At a critical point in the history of South Sudan, prior to its independence, assessments regarding gender inequitable norms and GBV were undertaken in seven sites during 2009–2011. The findings of this study reveal an acceptance of gender inequitable norms and an acceptance of violence against women within sampled communities in South Sudan. Both women and men were in agreement with gender inequitable norms and supported violence against women. While the study applied a Gender Equitable Men scale, the findings encourage further exploration of a Gender Equitable Women scale in South Sudan. A gender equitable woman could be defined as a woman who believes that women and men should have equal rights and opportunities, believes that women and men share responsibility in decisions and roles in the household and community, and who would be opposed to violence against women. Further research to understand gender inequality and GBV in South Sudan are needed to inform programming and policies. Achievement of gender equality and the elimination of violence against women are central to country development and to the health of communities in South Sudan. South Sudan has the unique opportunity, as the world’s newest nation, to promote gender equality and to empower women and girls in its communities.

## Abbreviations

ARC: American Refugee Committee; CPA: Comprehensive Peace Agreement; GBV: Gender-based Violence; GEM: Gender Equitable Men (Scale); IPV: Intimate Partner Violence; SHHS: Sudan Household Health Survey

## Competing interests

The co-authors declare no competing interests.

## Authors’ contributions

JS contributed to study design, data acquisition, and data entry; directed data analysis; interpreted data; and wrote the initial draft of the manuscript. SA assisted in data interpretation and writing of the manuscript. AMM conducted the data analysis and contributed to data interpretation and writing of the manuscript. MH assisted in study design, supervised data analysis, and contributed to data interpretation and writing of the manuscript. SC and DS contributed to study design, data acquisition, data interpretation, and writing of the manuscript. MM supported the project as Monitoring and Evaluation officer and provided expertise for data interpretation and manuscript writing. PP assisted with data interpretation and manuscript writing. All authors critiqued drafts of the paper and JS was responsible for final collation of inputs and redrafting. All authors read and approved the final manuscript.
